# Proinflammatory keratinocytes drive a novel mouse model of autoimmunity with systemic and cutaneous lupus erythematosus

**DOI:** 10.1007/s44466-025-00024-y

**Published:** 2026-02-03

**Authors:** Jingru Tian, Liqing Shi, Dingyao Zhang, Xu Yao, Jun Lu, Ming Zhao, Qianjin Lu

**Affiliations:** 1 Institute of Dermatology, Chinese Academy of Medical Sciences and Peking Union Medical College, Nanjing, China; 2 Key Laboratory of Basic and Translational Research On Immune-Mediated Skin Diseases, Chinese Academy of Medical Sciences, Nanjing, China; 3 Jiangsu Key Laboratory of Molecular Biology for Skin Diseases and STIs, Nanjing, China; 4 Department of Allergy and Rheumatology, Hospital for Skin Diseases, Institute of Dermatology, Chinese Academy of Medical Sciences and Peking Union Medical College, Nanjing, China; 5 Program of Computational Biology and Bioinformatics, Yale University, New Haven, CT USA; 6 Yale Stem Cell Center, New Haven, CT USA; 7 Department of Genetics, Yale University School of Medicine, New Haven, CT USA; 8 Yale Cancer Center, New Haven, CT USA; 9 Yale Center for RNA Science and Medicine, New Haven, CT USA; 10 Yale Cooperative Center of Excellence in Hematology, New Haven, CT USA

**Keywords:** Lupus erythematosus, Mice model, Keratinocytes, Peroxisome proliferator-activated receptor gamma, Dendritic cells

## Abstract

The pathogenesis of autoimmune diseases remains poorly understood, largely because existing models fail to capture both the initial triggers and the full spectrum of systemic manifestations. Here, we identify the skin epithelium as an initiating site of autoimmune activation. Specifically, we found that PPARγ levels are broadly reduced in basal-layer keratinocytes from patients across the lupus disease spectrum. To investigate the functional impact of this epithelial defect, we employed keratinocyte-specific gene editing in mice. Localized editing induced cutaneous lupus-like inflammation, while more extensive epithelial perturbation triggered rapid systemic autoimmunity, characterized by multi-organ inflammation and autoantibody production. Moreover, ultraviolet exposure accelerated the progression from cutaneous to systemic disease, recapitulating the spectrum transition and clinical photosensitivity. Mechanistically, we found that proinflammatory keratinocytes promoted the emergence of migratory CCR7⁺ dendritic cells, which appeared to initiate and amplify immune activation beyond the epithelial niche. Our findings establish the skin epithelium as a critical initiator of autoimmunity and provide a tunable model that recapitulates key features of human lupus spectrum.

**Supplementary Information:**

The online version contains supplementary material available at 10.1007/s44466-025-00024-y.

## Introduction

Lupus spectrum diseases including systemic lupus erythematosus (SLE) and cutaneous lupus erythematosus (CLE) are autoimmune diseases characterized by the loss of immune tolerance and the manifestation of inflammation in skin or multiple organs [1, 2]. Animal models are essential for unraveling disease triggers and progression. However, modeling the full spectrum of lupus in mice has remained a major challenge.

Most existing models concentrate on the systemic aspect of the disease, including NZB/W F1 [3], MRL/lpr [4], BXSB/Yaa [5], and PD-1H-deficient mice [6], develop multi-organ inflammation and autoantibodies, but rarely recapitulate the cutaneous pathology or photosensitivity in human disease [7]. Conversely, models that reproduce CLE features, such as xenotransplantation of SLE patient immune cells with ultraviolet (UV)-induced lesions [8], do not transition to systemic disease. Even the widely used MRL/lpr model, although it displays both skin and systemic involvement, is limited by mutations in Fas that do not parallel human SLE genetics [9], low penetrance (~20%) and atypical ulcerative lesion morphology [10], as well as a complex breeding scheme that complicates additional genetic modification [11]. Thus, the lack of an in vivo system that recapitulates both cutaneous involvement and systemic disease progression has limited our mechanistic understanding of lupus spectrum pathogenesis.

Peroxisome proliferators-activated receptor (PPAR)γ, a crucial regulator of lipid metabolism, has gradually attracted attention in the studies of SLE. Germline *Pparg* knockout mice develop autoimmune symptoms in the kidney [12]. However, the disruption of PPARγ function in all bodily cells in human is rare and therefore the germline *Pparg* loss cannot represent a triggering event in the vast majority, if not all, of human cases. Similarly, knockout of *Pparg* in myeloid cells via Lys2-cre in mice can also cause autoimmune glomerulonephritis [13, 14], but there is no evidence that human SLE patients have less PPARγ expression in myeloid cells. In fact, one study reported an opposite change, where CD14^+^ monocytes of SLE patients show higher PPARγ expression [15].

Our study initiated from our discovery of a pronounced reduction of PPARγ protein expression within the basal layer keratinocytes of SLE and CLE skin lesions compared to healthy individuals and those with other inflammatory skin conditions [16]. Localized keratinocyte-specific depletion of *Pparg* in mice induced rapid disease onset and produced cutaneous lupus–like inflammation, whereas broader epithelial involvement triggered systemic autoimmunity characterized by multi-organ inflammation and circulating autoantibodies. These mice exhibited photosensitivity, and UV exposure accelerated the transition from skin-confined disease to systemic involvement, mirroring clinical progression. Moreover, we observed enhanced activation and migratory potential in dendritic cells (DCs) from lupus skin lesions, suggesting that proinflammatory keratinocytes license DCs to propagate immune activation beyond the epithelial niche. Collectively, these findings identify the skin epithelium as an initiating site of autoimmune activation and demonstrate that keratinocyte perturbation is sufficient to drive the transition from localized to systemic disease. Our study also provides a genetically tractable and rapidly inducible mouse model that recapitulates the lupus spectrum. This model serves as a powerful tool for dissecting the mechanisms underlying autoimmune initiation and progression.

## Results

### Characteristic reduction of PPARγ levels in basal layer keratinocytes of skin lesions in lupus spectrum diseases

**Fig. 1:**
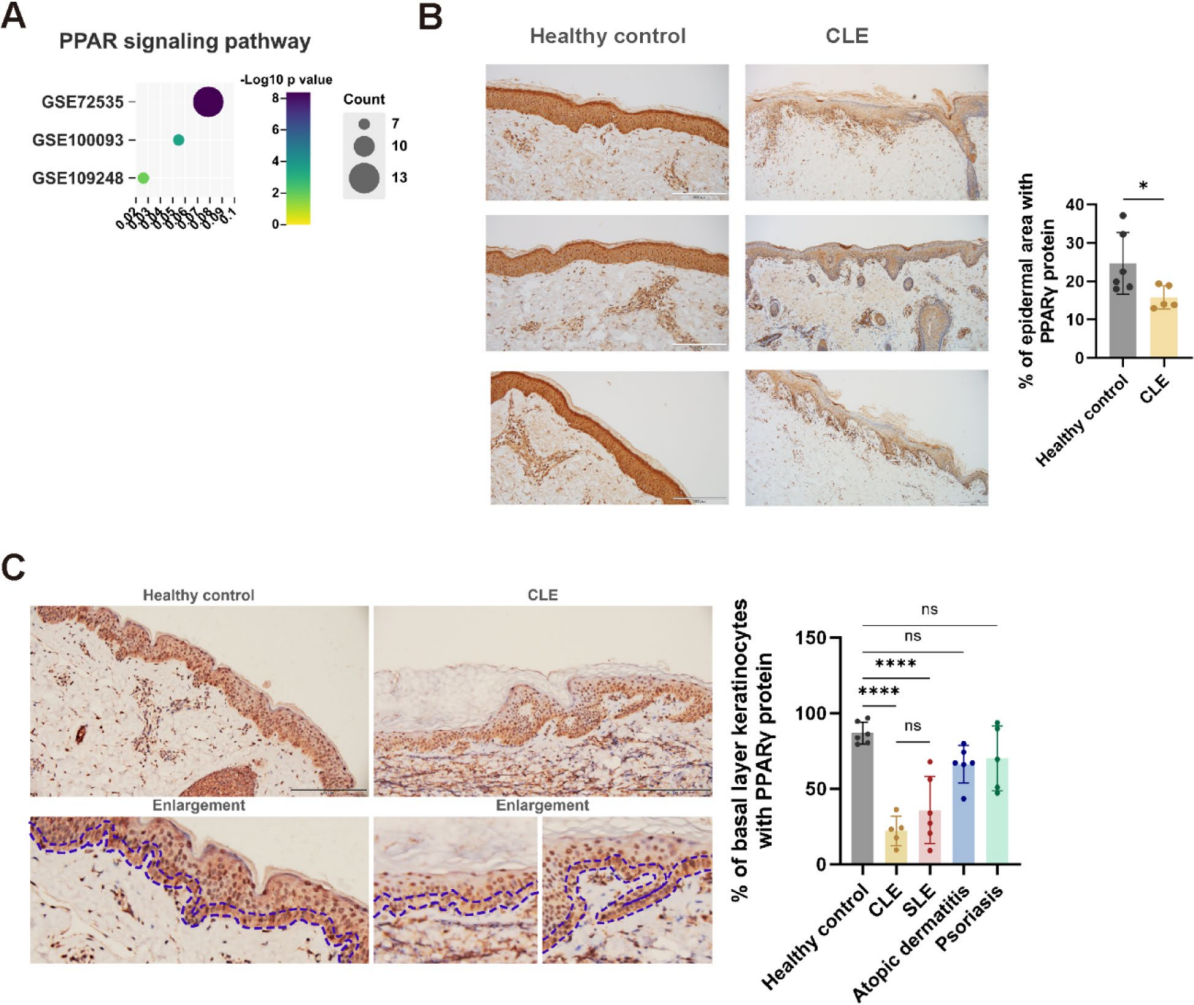
Decreased PPARγ in basal layer keratinocytes of SLE and CLE patients. **A** Transcriptome data from CLE skin lesions (GSE72535, GSE100093, and GSE109248) were re-analyzed. Differentially expressed genes (DEGs) between CLE lesions and healthy control skin were subjected to Gene Set Enrichment Analysis (GSEA) to assess PPAR pathway activity. The color scale indicates the statistical significance of enrichment (-Log10 p-value), with darker colors representing stronger significance. Dot size corresponds to the number of PPAR pathway-related genes among the differentially expressed genes. GSE72535: 13 genes enriched (7.9% of DEGs), *p* = 4.07e-09; GSE100093: 7 genes enriched (5.5% of DEGs), *p* = 0.00033; GSE109248: 7 genes enriched (2.6% of DEGs), *p* = 0.014. **B** PPARγ protein expression in the epidermis was visualized using immunohistochemistry (IHC), with representative images from healthy controls and CLE patients. The percentage of affected epidermal area is shown in the graph (*n* = 5-6). Scale bars represent 200 μm. **C** PPARγ protein expression in the basal layer of keratinocytes was visualized by IHC, showing representative samples from healthy controls and CLE patients. The basal layer of keratinocytes is indicated between two dark blue dotted lines in the magnified images. The percentage of basal layer keratinocytes expressing PPARγ is shown in the graph. Scale bars represent 200 μm. For all panels, the error bars represent the SDs, and the center values represent the means. Comparisons between two groups were performed using a two-tailed unpaired Student's t-test, and multiple group comparisons were analyzed using one-way ANOVA. *p < 0.05; **p < 0.01; ***p < 0.001; ****p < 0.0001; ns: not significant

Through analyzing published transcriptome GEO datasets of human CLE cutaneous lesions (GSE72535; GSE100093; GSE109248), we identified the significant downregulated PPAR pathway in CLE cutaneous lesions compared to normal skin (Fig. 1A). Further immunohistochemical analysis of skin tissues have revealed that protein content of PPARγ, a key molecule in the PPAR pathway, was significantly downregulated in keratinocytes of human CLE lesions (Fig. 1B). This finding parallels our recent observations in SLE skin lesions [16]. Moreover, unlike inflammatory lesions such as psoriasis and atopic dermatitis, which maintain certain levels of PPARγ in the basal layer of keratinocytes, SLE skin lesions show a reduction of PPARγ in these cells [16]. Similarly, in CLE lesions, PPARγ levels are notably decreased in the basal layer of keratinocytes (Fig. 1C, Table S1). This distinct phenotype, differing from that of inflammatory lesions, indicates a shared unique alteration in the basal layer keratinocytes across lupus spectrum diseases.

### Reduced basal layer keratinocyte-specific PPARγ drives the spontaneous development of SLE-like phenotype

To better replicate the conditions observed in lupus patients, we generated a lupus spectrum disease model by crossing immunocompetent *Pparg*^fl/fl^ mice on a C57BL/6 background with *Krt5*^creERT2/+^ mice, in which the expression of recombinant creERT2 protein can be induced in basal layer keratinocytes and other epithelial cells. The induction of cre-mediated conditional knockout (KO) was achieved by administering tamoxifen (TAM) intraperitoneally to *Pparg*^fl/fl^;*Krt5*^creERT2/+^ mice (*Pparg* KO mice). By day 11 post-injection, KO mice exhibited SLE-like phenotypes with 100% penetrance, characterized by severe cutaneous lesions, such as alopecia, erythema, scabbing, and ulceration (Fig S1A, B). Histopathological examination revealed hallmark features of lupus, including hyperkeratosis, follicular plugging, inflammatory cell infiltration, and vacuolar degeneration in the basal layer (Fig S1C). These pathological changes were accompanied by systemic manifestations, including proteinuria (Fig S1D), IgG deposition in the renal glomeruli (Fig S1E), and elevated serum autoantibodies (Fig S1F). In contrast, none of the control mice (C57BL/6 or *Pparg*^fl/fl^ mice) treated with or without TAM displayed any inflammatory skin lesions or systemic involvement.

**Fig. 2:**
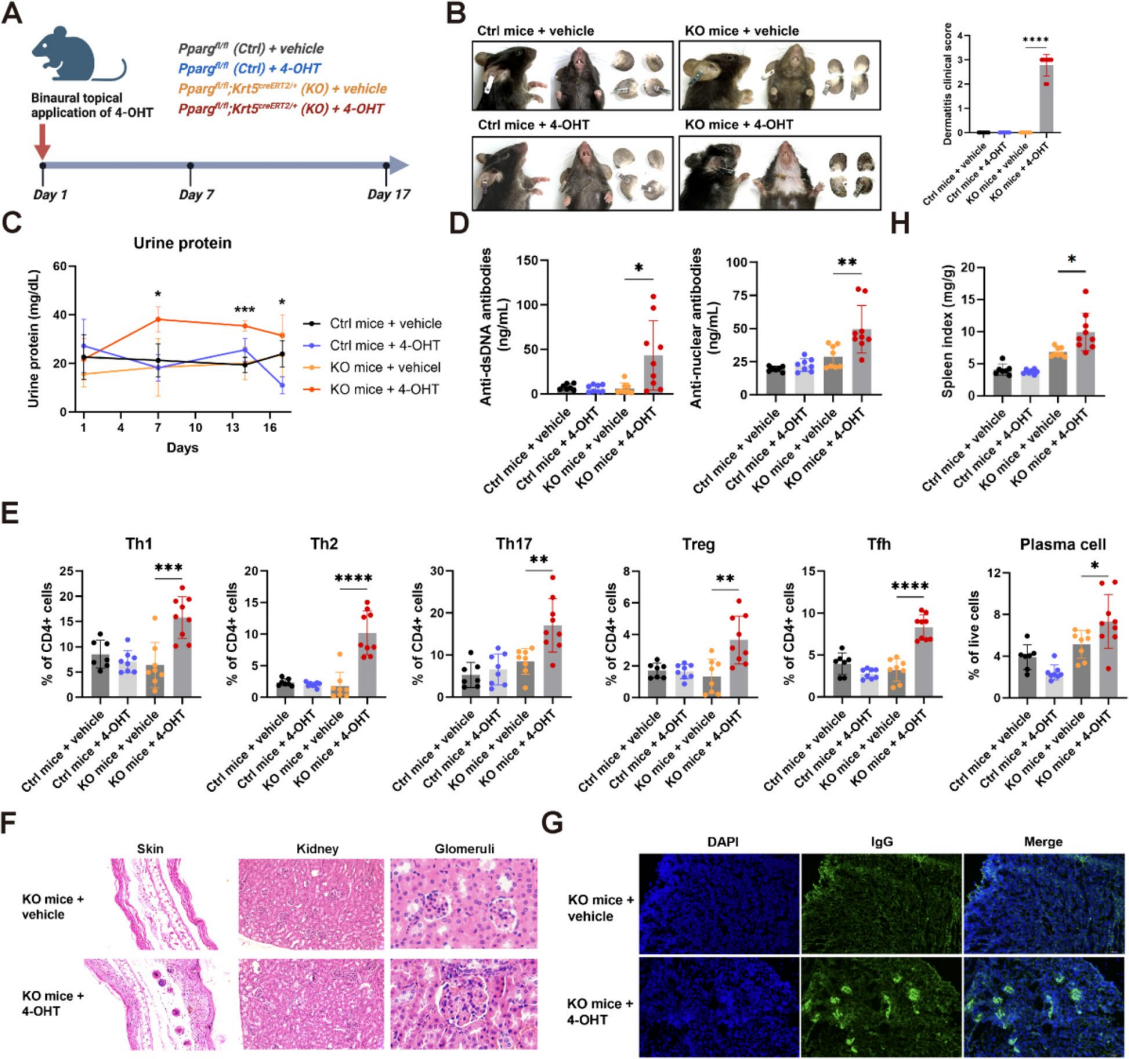
Reduced basal layer keratinocyte-specific PPARγ drives the spontaneous development of SLE-like phenotype. **A** Schematic illustration of the SLE model in which the level of keratinocyte-intrinsic PPARγ was decreased locally. 4-OHT or vehicle was administered topically to both ears from Day 1 to Day 5 to *Pparg*^fl/fl^ (Ctrl) and *Pparg*^fl/fl^; *Krt5*^creERT2/+^ (KO) mice. The kidney, skin and spleen were harvested on Day 17. **B** Representative images of mice from a representative experiment and graph showing dermatitis clinical scores (dermatitis clinical scoring assesses severity of lesions, including erythema, edema, scaling, and ulceration, using a 0-4 scale). Images were acquired on Day 17. **C** Urine protein levels were quantified on the indicated days (*n* = 7-9). **D** The levels of anti-double-stranded DNA antibodies (left) and antinuclear antibodies (right) in peripheral blood (*n* = 7-9). **E** Percentages of Th1, Th2, Th17, Treg and Tfh subsets among CD4^+^ T cells, and plasma cells among live cells in the spleen were analyzed (*n* = 7-9). **F** Histological analysis of ear skin, kidneys, and glomeruli. **G** Representative images of glomerular IgG deposition. **H** Graph showing spleen index (spleen weight [mg] to body weight [g] ratio) (*n* = 7-9). For all panels, the error bars represent the SDs, and the center values represent the means. Comparisons between two groups were performed using a two-tailed unpaired Student's t-test, and multiple group comparisons were analyzed using one-way ANOVA. *p < 0.05; **p < 0.01; ***p < 0.001; ****p < 0.0001. Data are representative of two or more independent experiments

Notably, these immunocompetent mice demonstrated a robust autoimmune response, often leading to mortality around Days 12 or 13. To more accurately mimic the localized nature of SLE skin lesions and restrict *Pparg* deletion to keratinocytes, we employed a refined strategy involving topical application of 4-hydroxytamoxifen (4-OHT) to both ears of the mice (Fig. 2A). Remarkably, all 4-OHT-treated keratinocyte-specific *Pparg* KO mice developed pronounced SLE-like phenotypes, including severe skin inflammation (Fig. 2B), proteinuria (Fig. 2C), and increased serum autoantibodies (Fig. 2D). Flow cytometry analysis revealed significant alterations in the immune cell profile, with increased proportions of regulatory T cells (Tregs), T helper 1 (Th1), T helper 2 (Th2), T helper 17 (Th17), and T follicular helper (Tfh) cells, as well as a marked expansion of plasma cells (Fig. 2E). These immune profile alterations closely parallel the dysregulation observed in human SLE. Histological analysis of skin lesions revealed typical lupus pathology, including acanthosis, hyperkeratosis, follicular plugging, lymphocytic infiltration, and mild interface dermatitis (Fig. 2F). Renal histology demonstrated moderate chronic multifocal to segmental glomerulointerstitial nephritis with significant IgG deposition in the glomeruli (Fig. 2F, G). Additionally, spleen index was significantly increased in the SLE model mice (Fig. 2H), further confirming systemic autoimmune involvement.

### The extended phenotype of keratinocyte conditional *Pparg* knockout SLE mice

**Fig. 3:**
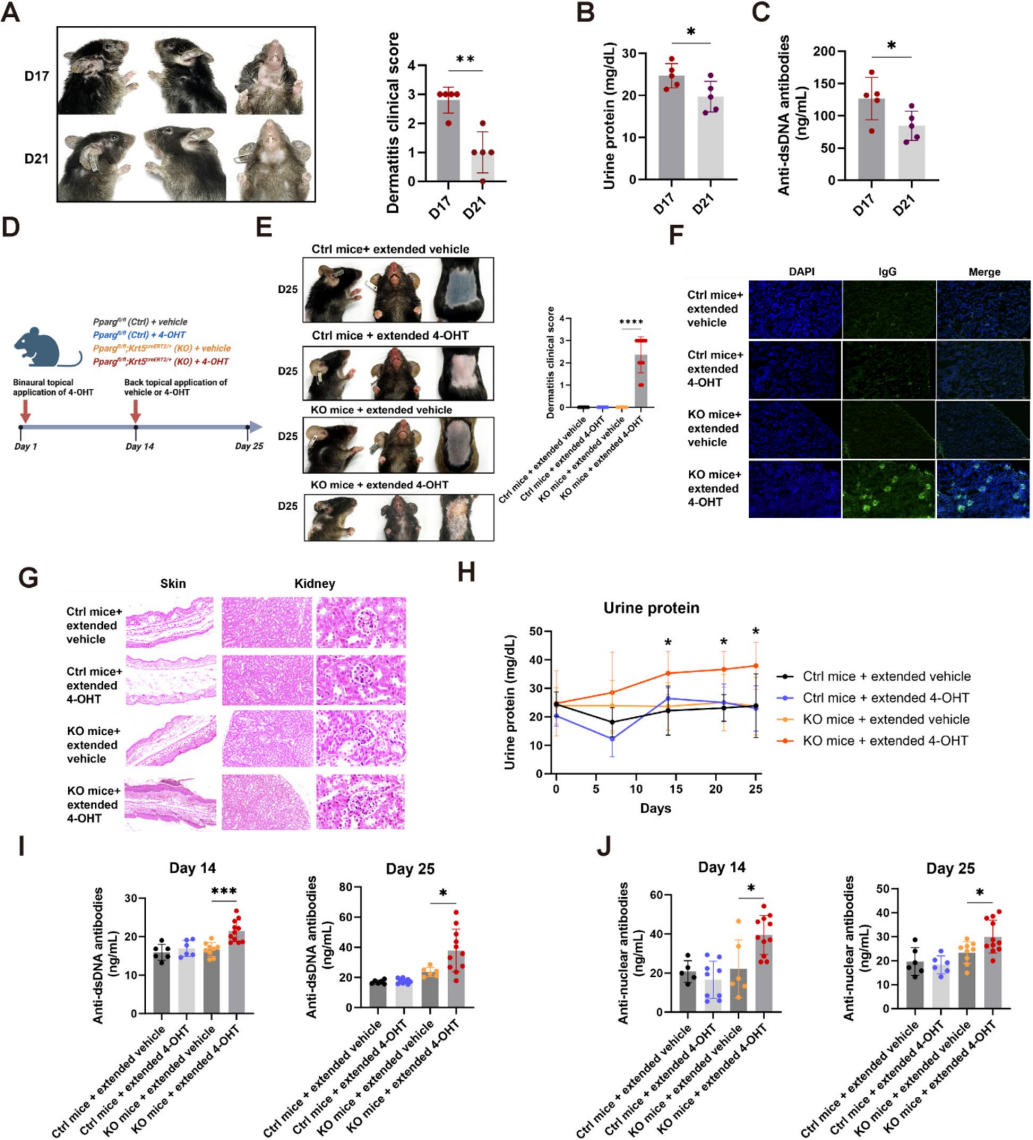
The extended phenotype of keratinocyte conditional *Pparg* knockout SLE mice. **A** Representative images showing gradual resolution of SLE-like skin lesions on Days 17 and 21, and graph showing dermatitis clinical scores. **B** Graph illustrating the progressive decline in urine protein levels on Days 17 and 21 (*n* = 5). **C** Graph showing reduction in anti-dsDNA antibody levels on Days 17 and 21 (*n* = 5). **D** Schematic of the experimental design for extending the SLE phenotype. **E** Representative images of treated mice on Day 25 and graph showing dermatitis clinical scores. **F** Representative images of glomerular IgG deposition on Day 25. **G** Histological analysis of ear skin, kidneys, and glomeruli on Day 25. **H** Urine protein levels measured on indicated days (*n* = 6-11). **I** Quantification of anti-double-stranded DNA antibodies in peripheral blood (*n* = 6-11) on Days 14 and 25. **J** Quantification of antinuclear antibodies in peripheral blood (*n* = 6-11) on Days 14 and 25. For all panels, the error bars represent the SDs, and the center values represent the means. Comparisons between two groups were performed using a two-tailed unpaired Student's t-test, and multiple group comparisons were analyzed using one-way ANOVA. *p < 0.05; **p < 0.01; ***p < 0.001; ****p < 0.0001. Data are representative of two or more independent experiments

Intriguingly, in keratinocyte PPARγ-deficient mice, the SLE-like phenotype began to improve by Day 21, with signs of reduced skin lesions, lower urinary protein levels, and decreased anti-dsDNA antibody concentrations (Fig. 3A-C). These amelioration of symptoms in the mouse model may resemble the remission process observed in human patients.

To extend the lifespan of the typical SLE-like phenotype, thus offering a more suitable tool for subsequent treatment screening, we initiated an additional back skin application of 4-OHT on Day 14 for 5 consecutive days to further suppress PPARγ protein expression in keratinocytes (Fig. 3D). Post-re-administration, we observed a prolonged SLE-like phenotype, with mice maintaining significant skin lesions, urinary protein, and elevated autoantibodies on both Days 14 and 25 (Fig. 3E-J). This model featuring an extended phenotype provides a stable and typical SLE-like manifestation for over 25 days, thereby allowing for an ample observation period for efficacy testing.

### Reduction of PPARγ in basal layer keratinocytes with limited area drives spontaneous development of CLE-like phenotype

**Fig. 4:**
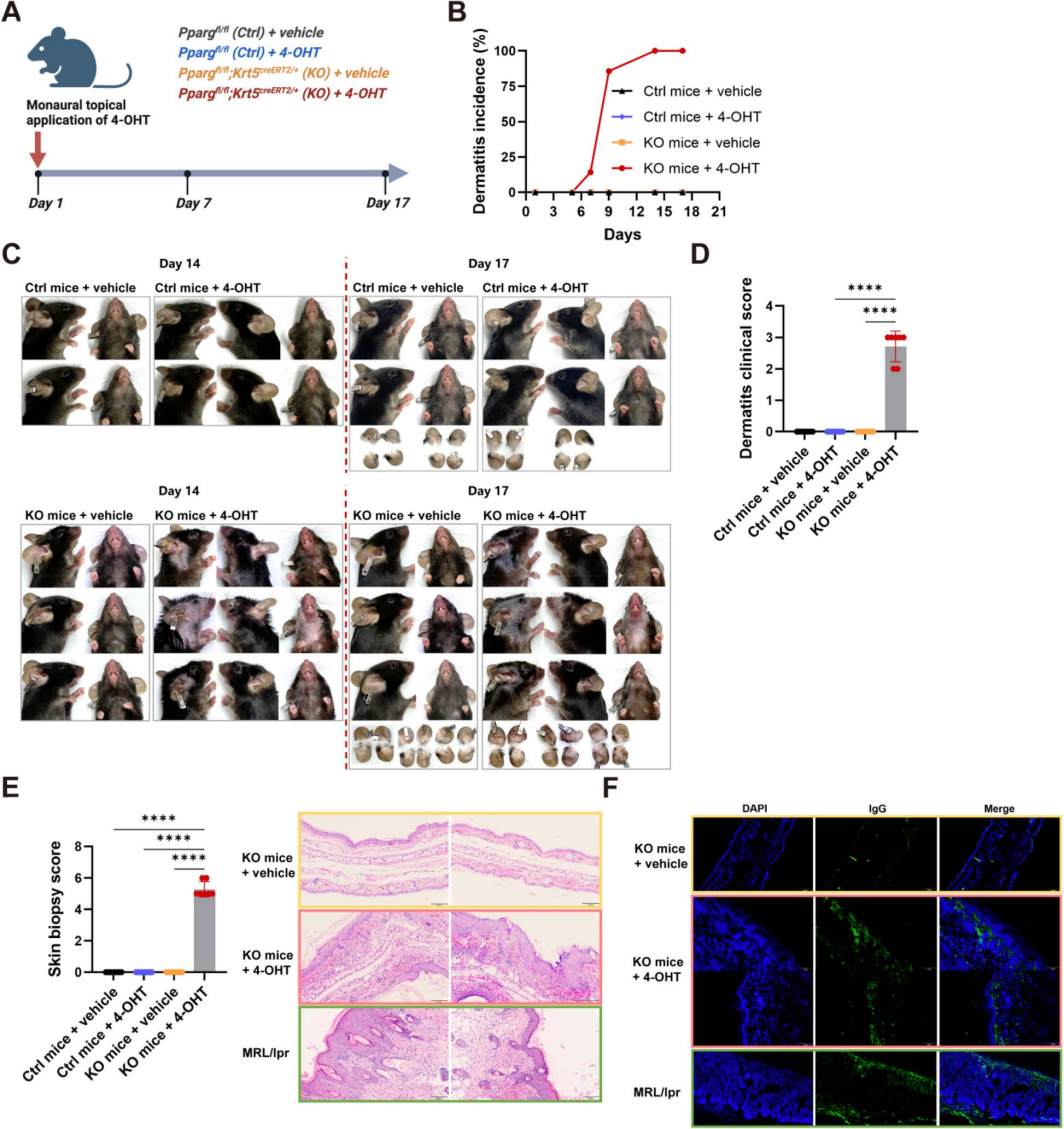
Reduction of PPARγ in basal layer keratinocytes with limited area drives spontaneous development of CLE-like phenotype. **A** Schematic illustration of the SLE model in which the level of keratinocyte-intrinsic PPARγ was decreased locally. 4-OHT or vehicle was administered topically to single ear from Day 1 to Day 5 to *Pparg*^fl/fl^ (Ctrl) and *Pparg*^fl/fl^;*Krt5*^creERT2/+^ (KO) mice. The kidney, skin and spleen were harvested on Day 17. **B** Temporal analysis of the incidence of dermatitis in murine models (*n* = 6-7). **C** Representative images of treated mice on Day 14 and Day 17. **D** Graph of dermatitis clinical scores on Day 17 (*n* = 6-7). **E** Graph of histopathological scores on Day 17, with representative images of histological examination of ear skin (*n* = 6-7). **F** Representative images of IgG deposition in the skin basement membrane zone. For all panels, the error bars represent the SDs, and the center values represent the means. Comparisons between two groups were performed using a two-tailed unpaired Student's t-test, and multiple group comparisons were analyzed using one-way ANOVA. *p < 0.05; **p < 0.01; ***p < 0.001; ****p < 0.0001. Data are representative of two or more independent experiments

The extent area of *Pparg* deletion in keratinocytes was closely associated with disease severity. When the application of 4-OHT was confined to a smaller area, such as a single ear, the KO mice predominantly exhibited CLE-like skin lesions with barely any systemic involvement (Fig. 4A). By Day 10, mice in the 4-OHT group showed persistent erythema and atrophic plaques with scaling at the site of 4-OHT application, as well as extensive erythema affecting the ear, jaw, and chest, whereas vehicle-treated controls displayed no visible cutaneous lesions (Fig. 4B-D). Histopathological evaluation of the cutaneous lesions revealed CLE-like characteristics, including hyperkeratosis, follicular plugging, inflammatory cell infiltration, and basal layer vacuolar changes (Fig. 4E). The ear lesions in 4-OHT treated mice closely resembled those seen in the MRL/lpr lupus model, as distinct linear and granular IgG deposits along the basement membrane zone, which were absent in vehicle-treated mice (Fig. 4F).

Despite the cutaneous manifestations, these CLE-like mice exhibited only a modest, non-significant reduction in body weight from Day 7 to Day 14 (Fig S2A) and did not develop proteinuria (Fig S2B). Moreover, renal histology revealed normal glomerular morphology with no immune complex deposition, distinct from the typical renal pathology seen in the MRL/lpr model (Fig S2C). Immunofluorescence analysis confirmed the absence of IgG deposition in the glomeruli of CLE-like mice, similar to vehicle-treated controls (Fig S2D). Additionally, serum levels of anti-double-stranded DNA antibodies and anti-nuclear antibodies remained unaltered (Fig S2E), and spleen index showed no significant increase (Fig S2F).

Overall, we successfully established a robust lupus spectrum disease model in immunocompetent C57BL/6 mice by targeting *Pparg* in keratinocytes. The disease phenotype in this model can be precisely regulated by modifying the area extent of keratinocyte-specific gene editing.

### Keratinocyte conditional *Pparg* knockout SLE mice with extended phenotype as a potential platform for therapeutic screening

We then introduced an intraperitoneal injection of cyclophosphamide, the commonly used SLE treatment drug to SLE mice model to explore its potential for therapeutic screening (Fig S3A). Following the cyclophosphamide injection, the SLE model mice exhibited less severe skin lesions, reduced proteinuria, lower serum autoantibody levels, and decreased antibody deposition in the skin basement membrane zone as well as renal glomeruli (Fig S3B-F). This indicates the potential efficacy of using these SLE-like phenotype mice as a platform for therapeutic screening.

### Keratinocyte conditional *Pparg* knockout CLE mice model as tools for evaluating treatment efficacy

We topically applied corticosteroids to the ears of the mice and monitored the therapeutic effect of CLE conventional treatment on their cutaneous lesions (Fig S4A). As we expected, topical corticosteroids significantly improved mice ear erythema, desquamation, and ulceration, as well as facial alopecia, erythema, and desquamation in mice (Fig S4B, C). Histopathology showed that topical glucocorticoids reduced epidermal thickness, relieved hair follicle angle plugs, inflammatory cell infiltration, and IgG deposition in the basement membrane zone of mice (Fig S4D-F). These findings suggest that topical glucocorticoid application effectively ameliorates the skin lesions of the model mice similar to CLE patients. Throughout the entire treatment phase, the mice displayed no significant alterations in body weight, urine protein, and autoantibodies (Fig S4G-I). This indicates that our CLE mouse model is capable of consistently preserving the CLE phenotype without systemic complications during the screening for treatment efficacy, and can serve as a preclinical screening platform for CLE therapeutics.

### UV irradiation exacerbates CLE and potentially facilitates its transition to SLE

**Fig. 5:**
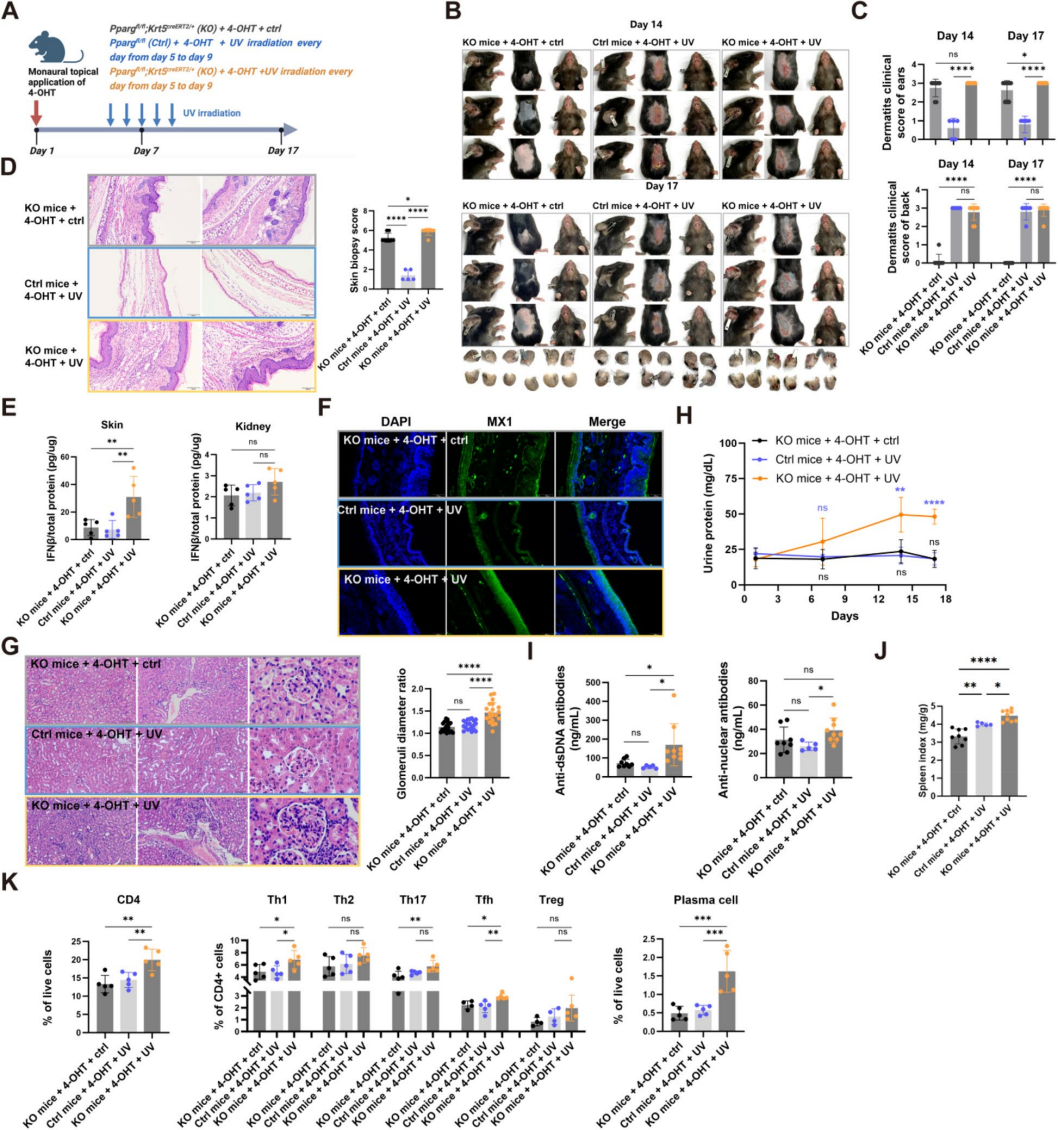
UV irradiation exacerbates CLE and potentially facilitates its transition to SLE. **A** Schematic of the experimental design illustrating the conversion of CLE to SLE facilitated by UV exposure. After topical application of 4-OHT to a single ear of *Pparg*^fl/fl^ (Ctrl) and *Pparg*^fl/fl^;*Krt5*^creERT2/+^ (KO) mice, UV exposure or control treatment was applied to the exposed skin for five consecutive days. **B** Representative images of treated mice. **C** Graph showing dermatitis clinical scores for the ear and back. **D** Representative histological images of ear skin. **E** IFNβ/total protein levels in skin and kidney tissues detected by ELISA (*n* = 5). **F** Representative images of MX1 immunofluorescence staining in mouse skin. **G** Representative histological images of kidneys and glomeruli, along with a graph showing glomerular diameter ratio. **H** Urine protein levels quantified on indicated days (*n* = 7-9). **I** Levels of anti-double-stranded DNA antibodies (left) and antinuclear antibodies (right) in peripheral blood (*n* = 7-9). **J** Graph showing spleen index (*n* = 7-9). **K** Analysis of the percentages of CD4^+^ T cells among live cells (left), and the percentages of Treg, Th1, Th2, Th17, and Tfh subsets among CD4^+^ T cells (middle), and plasma cells among live cells (right) in the spleen (n = 4-5). For all panels, the error bars represent the SDs, and the center values represent the means. Comparisons between two groups were performed using a two-tailed unpaired Student's t-test, and multiple group comparisons were analyzed using one-way ANOVA. *p < 0.05; **p < 0.01; ***p < 0.001; ****p < 0.0001; ns: not significant. Data are representative of two or more independent experiments

Lupus patients exhibit significant photosensitivity, UV irradiation has been associated with flare-ups, and can promotes the cutaneous to systemic transformation of spectrum diseases. Prior models of SLE with cutaneous manifestations or artificially induced CLE model have fallen for recapitulating the clinical course of the disease in patients, and thus, fail to faithfully replicate the state of CLE progression. Our CLE model, however, offers a satisfied tool for investigating the pathological processes inherent to CLE. To this end, we subjected our CLE mouse model to UV irradiation to simulate sunlight's impact on the disease state of CLE (Fig. 5A).

UV irradiation led to skin damage at the exposed areas in both normal mice with intact keratinocyte-PPARγ and CLE-affected mice (Fig. 5B, C). Additionally, in CLE mice, UV exposure not only worsened ear skin lesions (Fig. 5B-D) but also increased type I IFN production (Fig. 5E, Fig S5) and activated the type I IFN signaling in the local skin lesions (Fig. 5F). Furthermore, UV irradiation hastened the systemic progression of CLE in these mice, as evidenced by the onset of proteinuria (Fig. 5H), a rise in autoantibodies (Fig. 5I), spleen enlargement (Fig. 5J), and changes in immune cell types (Fig. 5K). These observations suggest that although CLE typically starts as a localized skin issue, it can evolve into systemic changes. The local overproduction of type I IFN and the sensitization of lymphocytes might lay the foundation for this systemic involvement [17].

### Skin infiltrating DCs are over-activated in lupus spectrum diseases

Our murine model demonstrated a dynamic transition from CLE to SLE. To further elucidate the pivotal factors responsible for the transformation of CLE, which typically affects only local skin tissues, to systemic SLE involvement, we analyzed single-cell transcriptomic data from skin lesions in Discoid lupus erythematosus (DLE) and SLE patients [17]. The unique phenotype of DCs attracted our attention.

**Fig. 6:**
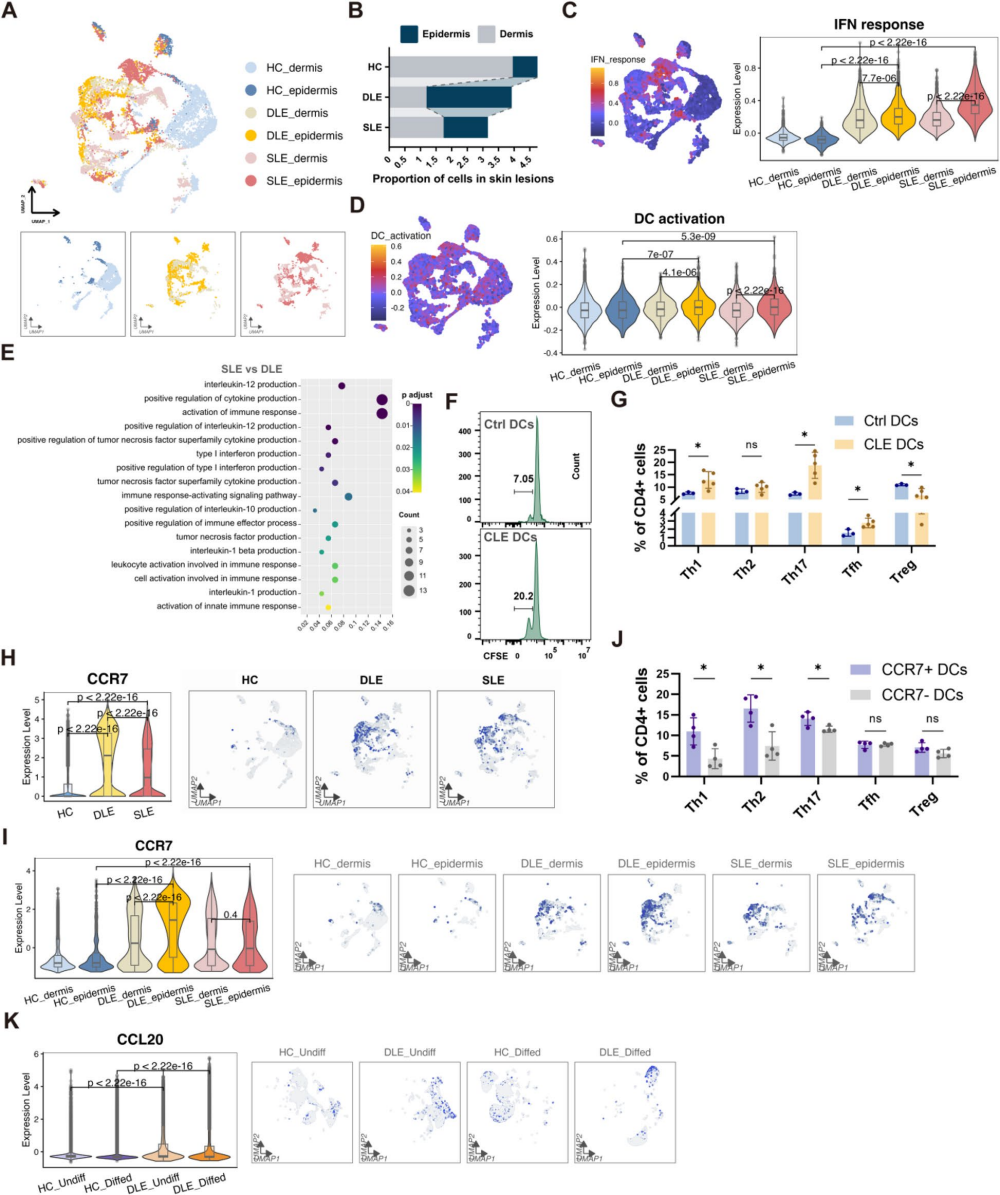
CCR7^+^ DCs from lupus lesions promote proliferation and pro-inflammatory transformation of T cells. **A-D** DCs identified in UMAP plots (**A**), Proportion of DCs (**B**), IFN response in DCs (**C**) and activation status of DCs (**D**) in the epidermis and dermis of healthy controls, DLE, and SLE patients. **E** Pathway enrichment analysis of upregulated genes that differentially expressed in SLE skin lesions compared to DLE lesions. **F** CFSE assay demonstrating the proliferative effect of DCs derived from CLE skin on CD4^+^ naive T cells. **G** The proportions of Th1, Th2, Th17, Tfh, and Treg cells were evaluated following a 5-day co-culture of skin-derived DCs from Ctrl or CLE mice, with CD4^+^ naive T cells. **H-I** Violin plots (left panel) and UMAP plots (right panel) illustrating the CCR7 gene expression in DCs from skin (**H**), epidermis and dermis (**I**) in healthy controls, DLE, and SLE patients. **J** The proportions of Th1, Th2, Th17, Tfh, and Treg cells were evaluated following a 5-day co-culture of CCR7^+^ or CCR7^−^ skin-derived DCs from lupus mice, with CD4^+^ naive T cells. **K** Violin plots (left panel) and UMAP plots (right panel) illustrating the CCL20 expression in undifferentiated keratinocytes from the skin of healthy controls and DLE patients. For all panels, the error bars represent the SDs, and the center values represent the means. Comparisons between two groups were performed using a two-tailed unpaired Student's t-test, and multiple group comparisons were analyzed using one-way ANOVA. *p < 0.05; ns: not significant. Data are representative of two or more independent experiments

DCs are the pivotal regulators of early immune responses in localized skin inflammation. In DLE skin lesions, we observed an evident increase in non-Langerhans DCs in the epidermis but a marked reduction in the dermis compared to healthy skin (Fig. 6A, B). Interestingly, this peculiar redistribution and reprogramming of DCs were also observed in SLE lesions with extensive systemic involvement (Fig. 6A, B). DCs from epidermis of both SLE and DLE lesions exhibited far stronger IFN-related signaling and cell activation signaling than their healthy counterparts and DCs in dermis (Fig. 6C, D). Overall, the enrichment of epidermal DCs and their heightened activation responses underscore the significant inflammatory alterations in lupus skin lesions, particularly in the epidermal microenvironment. Notably, DCs in SLE lesions demonstrated heightened immune activation and cytokine production capabilities compared to those in DLE lesions (Fig. 6E).

DCs were co-cultured with carboxyfluorescein succinimidyl ester (CFSE)-labeled T cells. Compared to those co-cultured with Ctrl-DCs, T cells co-cultured with CLE-DCs showed increased proliferation (Fig. 6F). Furthermore, CLE-DCs were able to effectively activate T cells to differentiate into effector T cells (Teff) rather than Tregs compared to Ctrl-DCs (Fig. 6G). This suggests that DCs in CLE skin lesions are effective in activating T cells and triggering downstream immune responses.

### CCR7^+^ DCs from lupus lesions promote proliferation and pro-inflammatory transformation of T cells

Interestingly, we found that DCs infiltrating SLE and DLE lesions expressed higher CCR7 than DCs in healthy skin (Fig. 6H). This CCR7^+^ DCs were mainly concentrated in the epidermis rather than the dermis of lupus (Fig. 6I). As a distinctive feature of mature DCs, CCR7 is an essential receptor that mediates the lymph node migration of DCs, and plays a pivotal role in the body's defense against pathogens and in maintaining immune tolerance to self-antigens [18]. Notably, CCR7^+^ DCs had a significantly enhanced ability to expand immunostimulatory Teff cells compared to CCR7^−^ DCs (Fig. 6J), suggesting CCR7^+^ DCs enriched in both SLE and CLE lesions are effectively poised to initiate downstream adaptive immune responses.

Epithelial tissues recruit DCs through the CCR6/CCL20 axis [19]. Keratinocytes in the DLE epidermis highly express CCL20 (Fig. 6K), which may be the key reason for the enrichment of DCs in the epidermis.

In summary, we have presented a spontaneously immunocompetent lupus spectrum diseases mouse model that closely mimics the clinical characteristics of lupus patients and simulates the dynamic progression from CLE to SLE, thereby furnishing an ideal platform for drug screening and pathological investigations. Furthermore, we uncovered the critical modulatory role of skin infiltrating DCs in the pathogenesis of lupus spectrum disorders, offering novel perspectives on the transformation and therapeutic approaches for these diseases.

## Discussion

Lupus is widely recognized as a polygenic disease influenced by both environmental and hormonal factors. With the rise of large-scale GWAS studies, over 270 candidate genes have been identified as being associated with lupus [20]. However, despite identifying key susceptibility genes such as HLA-DRB1, IRF5, STAT4, and TNFAIP3, the development of lupus animal models that accurately mimic patient phenotypes based on single-gene mutations remains elusive [21]. This highlights the likelihood that genetic variations contribute to disease progression through cumulative effects, where certain alleles exert greater influence as the overall genetic risk burden increases. Moreover, the absence of robust single- or multi-gene-driven lupus animal models underscores the critical role of non-genetic factors in disease onset and progression [22]. Prior studies have implicated PPARγ deficiency in immune cells may promote lupus-associated inflammation [15, 23, 24]. While in our lupus spectrum model, the reduction of PPARγ is not a germline mutation but is restricted to basal keratinocytes, suggesting that this alteration may result from non-genetic regulatory mechanisms. The model’s replication of the photosensitivity observed in lupus patients further reinforces the importance of both genetic factors and environmental triggers in disease onset.

Downregulation of PPARγ in non-cutaneous tissues has been linked to immune cell dysregulation, activation, and metabolic imbalance (15,25–27). However, the immunoregulatory function of PPARγ may be independent of its transcriptional activity. We found that PPARγ suppresses keratinocyte-derived type I IFN production through protein–protein interactions rather than transcriptional regulation. Loss of PPARγ protein rapidly breaks systemic immune tolerance and triggers SLE development [16]. The disease-modifying effects of PPARγ agonists appear to be limited, although in vitro studies have shown that PPARγ agonists suppress immune responses in peripheral blood mononuclear cells (PBMCs) from SLE patients [25], in vivo murine studies have demonstrated that PPARγ activation after the onset of systemic inflammation does not significantly prevent disease progression [15, 24]. Consistently, clinical trials with oral pioglitazone showed improvement in metabolic and vascular parameters but not in overall disease severity [26]. Therefore, in the absence of direct evidence demonstrating that PPARγ activation in immune cells halts disease progression in SLE, the therapeutic potential of PPARγ agonists should be interpreted with caution.

A noteworthy aspect of our model is that it does not exhibit the female bias typically seen in lupus patients. In both male and female mice, partial knockout of *Pparg* in basal keratinocytes led to disease onset with 100% penetrance, suggesting that sex differences might influence *Pparg* reduction at an upstream regulatory level. Future investigations into the differential roles of PPARγ in male and female mouse cells will be crucial for understanding this situation.

In our previous study, we identified excessive activation of DCs within lupus lesions, which migrated to the draining lymph nodes and promoted the differentiation of local T cells, thereby exacerbating the progression of SLE [16]. However, immune tolerance disruption is a multifaceted process. Beyond DCs, our single-cell analysis indicates that other cell types, such as hyperactivated fibroblasts and T cells within the lesions, could also contribute to disease onset. Notably, persistent inflammatory responses have been detected even in clinically normal skin during inflammatory skin disease remission [27]. The widespread deposition of IgG along the basement membrane zone in the clinically normal skin of SLE patients also indicates that the skin can mask ongoing pathological processes. While there has been little research on changes in clinically normal skin in lupus patients, our mouse model suggests that significant immune reprogramming and immune cell accumulation occur in preclinical skin. This points to a covert breakdown of immune tolerance, wherein the triggering factors may be masked by a robust pro-inflammatory phenotype by the time clinical symptoms manifest.

The complex interplay between SLE and CLE, particularly the pathophysiological mechanisms underlying disease progression from CLE to SLE, continues to pose significant challenges in lupus research. Our experimental data demonstrated conserved patterns of DC re-distribution and phenotypic reprogramming in cutaneous lesions across both SLE and CLE murine models, providing additional evidence for a potential common etiological pathway. Of particular significance, our comparative analysis revealed enhanced immunostimulatory capacity of DCs in SLE lesions relative to their CLE counterparts, suggesting a pivotal role in the transition from localized to systemic autoimmunity within the lupus disease continuum. Molecular characterization identified elevated CCL20 expression in CLE-derived epidermal keratinocytes, potentially explaining the compartmentalized DC accumulation observed in cutaneous lesions. This spatial restriction of activated DCs within the epidermal microenvironment may represent a natural barrier against systemic disease dissemination, maintaining disease activity within cutaneous boundaries. While these findings advance our understanding of disease mechanisms in lupus spectrum disorders, the precise molecular determinants of disease progression and the fundamental nature of SLE-CLE pathogenesis remain to be fully elucidated, warranting further mechanistic investigations.

In conclusion, we have developed a novel lupus spectrum disease model using immunocompetent C57BL/6 mice that closely mimics the complex clinical features and chronic course of lupus seen in patients. The phenotype of this model maintained longer than 10 days, is easy to breed, and addresses many limitations of previous models, such as complex genetic backgrounds, phenotypic differences from patients, immune deficiencies, difficulties in model establishment, low success rates, long modeling times, and high disease heterogeneity [28]. It provides a more accessible and reliable tool for lupus research. Most importantly, our model sheds new light on the role of keratinocyte homeostasis in driving systemic autoimmune responses and underscores the significance of tissue microenvironments in the progression of lupus. By refining our understanding of these processes, this model offers a useful platform for further investigation into the pathogenesis and treatment of lupus spectrum diseases.

## Methods

### Experimental design

The main purpose of this study is to explore the mechanism of lupus pathogenesis. The sample size of all the experiments was determined on the basis of our previous studies and prior experiments that were sufficient for statistical analysis, and considering animal welfare, as indicated in the figure legends. All experiments were performed at least two times or more independently. The mouse experiments were performed unblinded, as mice of different genotypes were identified and kept track of. The chemical treatment was designed according to the previously reported protocols as described in Materials and Methods or figure legends.

### Human subjects

To analyze PPARγ protein content in normal skin and cutaneous lesion of SLE and CLE, we collected skin tissue samples from patients and healthy controls (Table S1). The study protocol was approved by the Medical Ethics Committee in the Institute of Dermatology, Chinese Academy of Medical Sciences. All human subjects involved in this study were consented. Patients and the public did not contribute to the study design, implementation, reporting, or dissemination of the findings.

### Mice

All animal experiments were performed under an approved protocol by the Medical Ethics Committee in the Institute of Dermatology, Chinese Academy of Medical Sciences and following national and local governmental guidelines. C57BL/6J, *Pparg*^fl/fl^ (stock #NM-CKO-190071) and *Krt5*^creERT2/+^ (stock #NM-KI-190016) mice were purchased from the Shanghai Biomodel Organism Science & Technology Development Co. Ltd. The *Pparg*^fl/fl^ mice were bred with *Krt5*^creERT2/+^ mice to generate *Pparg*^fl/fl^;*Krt5*^creERT2/+^ keratinocyte conditional *Pparg* knockout mice. To confirm genotype, tail snips were collected at weaning (postnatal Day 21), and DNA was extracted using a standard alkaline lysis protocol.

For all experiments, 5- to 8-week-old mice were utilized. The mice were housed in the Animal Resources Center, which provided daily husbandry. In all animal studies, mice were randomly assigned to different groups, with the required sample size estimated based on initial pilot experiments. Blinding of the investigator was not implemented in this study. To minimize the potential impact of treatment order on the results, all animals were randomly assigned to experimental and control groups. Additionally, all measurements were conducted within the same time frame to ensure consistency. The locations of the animal cages were also randomized to mitigate interference from positional effects.

### Murine models

Figure S6 presents a schematic flowchart illustrating the experimental design for the CLE, SLE, and induced SLE models. To induce systemic Cre recombinase activation, tamoxifen (1mg per mouse, Sigma-Aldrich, #T5648) was dissolved in 5μl ethanol and 95μl corn oil (Beyotime, #ST1177) and administered intraperitoneally once daily for 5 consecutive days.

For localized keratinocyte-specific deletion of *Pparg*, 4-OHT (5mg/ml; Sigma-Aldrich, #H6278) was prepared in 10% DMSO and 90% corn oil and applied topically to the ears of *Pparg*^fl/fl^; *Krt5*^creERT2/+^ mice once daily for 5 days. Application of 40μl per day to both ears induced a SLE-like phenotype, whereas 20μl per day applied to a single ear produced a CLE-like phenotype.

To sustain the systemic autoimmune phenotype after initial induction, an additional course of topical 4-OHT (40μl 5mg/ml once daily for 5 days) was applied to the dorsal skin of *Pparg*^fl/fl^; *Krt5*^creERT2/+^ mice beginning on Day 14.

An approximately equal ratio of male and female mice (1:1) was used whenever possible. In experiments with limited gene-edited mice, sex distribution was balanced across groups.

### Monitoring of lupus phenotype

Throughout the course of the experiment, lupus phenotypes in the mice were closely monitored. Key indicators included the severity of skin lesions (such as erythema, alopecia, and ulceration), systemic symptoms (including weight loss and changes in activity levels), and signs of systemic involvement, such as proteinuria. Skin lesions were documented with photographs taken to track disease progression and therapeutic efficacy. Urine protein levels were assessed regularly using urine dipsticks, and serum levels of anti-double-stranded DNA antibodies and antinuclear antibodies were measured using enzyme-linked immunosorbent assays (ELISA). All data were quantitatively analyzed to evaluate disease progression and compare outcomes across experimental groups.

### Dendritic cells and CD4^+^ naive T cells co-culture

CD4^+^ naive T cells were isolated from mouse spleens using a MACS cell separation system (BioLegend #480040). For dendritic cells, we used flow cytometry to exclude potential contaminating cells, followed by magnetic bead sorting for CD11c-positive cells. Specifically, we gated out macrophages and monocytes using CD64 and Ly6C, Langerhans cells with CD207, neutrophils with Ly6G, T cells with CD3, NK cells with NK1.1, and B cells with CD19, thus removing non-dendritic immune cells. The remaining cells were then sorted using CD11c magnetic beads to obtain a purified and adequate population of DCs (Miltenyi #130-125-835).

In 96-well round-bottom plates, 2×10^5^ CD4^+^ T cells were co-cultured with 1×10^4^ dendritic cells, supplemented with 5μg/ml anti-CD3 (Invitrogen #16–0031-86) and 2μg/ml anti-CD28 (Invitrogen #16–0281-85) monoclonal antibodies. On the fifth day, cells were harvested for flow cytometric analysis to evaluate T-cell differentiation. For in vitro blocking assays, 10μg/ml of anti-mouse monoclonal antibodies were added.

### Cyclophosphamide and topical corticosteroid treatment

To investigate the efficacy of CTX in treating SLE mice model, 4-OHT was applied topically to both ears of *Pparg*^fl/fl^;*Krt5*^creERT2/+^ (KO) mice from Day 1 to Day 5. Subsequently, CTX (50 mg/kg, Sigma, C0768) or vehicle was administered via intraperitoneal injection on Days 6 and 13. For assessing the effectiveness of corticosteroid in treating CLE mice model, 4-OHT was topically applied to a single ear of *Pparg*^fl/fl^; *Krt5*^creERT2/+^ (KO) mice from Day 1 to Day 5, followed by topical application of corticosteroids (clobetasol propionate, 0.05%) to the same ear every two days starting from Day 10.

### Single-cell RNA sequencing of skin lesions

scRNA-seq was conducted following the guidelines provided in the Chromium Single Cell 3ʹ Reagent Kits v3.1 (10× Genomics, product codes: 1000268, 1000215, 1000120). Libraries were sequenced on an Illumina NovaSeq6000 System, targeting approximately 10,000 cells (ranging from 5000 to 12,000) per sample for single-cell RNA sequencing. Details regarding data processing and GO biological process analysis were outlined in our earlier studies [17].

Human skin scRNA-seq data were obtained from 14 epidermal single-cell suspensions (4 HC, 5 DLE, 5 SLE) and 16 dermal suspensions (4 HC, 5 DLE, 7 SLE), based on dataset GSE179633. By aggregating data from different samples, we generated summary profiles of the epidermal and dermal compartments under healthy, DLE, and SLE conditions. For each condition, the proportion of specific cell subtypes in the epidermis or dermis was calculated relative to the total number of skin cells, and the results are presented as percentages. Detailed information on cell counts and sequencing reads for each individual sample can be found in our previous study.

All analyses were conducted using standard computational pipelines for single-cell RNA sequencing data, with appropriate filtering to ensure high-quality results [17].

### Statistical analysis

Data were presented as mean ± standard deviation (SD). Statistical analyses were performed using GraphPad Prism 9. Student’s t-test was used for comparisons between two groups, and one-way or two-way ANOVA with post hoc Tukey’s test was used for multiple comparisons. A *p*-value of < 0.05 was considered statistically significant.

## Supplementary Information

Supplementary Material 1.

## Data Availability

All data supporting the findings of this study are included in the main text and Supplementary Information.
