# Supplementary material for: Proinflammatory keratinocytes drive a novel mouse model of autoimmunity with systemic and cutaneous lupus erythematosus
Source: Immun Inflamm. 2026 Feb 3;2(1):7. doi: 10.1007/s44466-025-00024-y (PMC12864243; doi:10.1007/s44466-025-00024-y)
Supplement: Supplementary file 1 — Supplementary Material 1. [file 44466_2025_24_MOESM1_ESM.docx]

Supplementary Materials for

**Proinflammatory Keratinocytes Drive a Novel Mouse Model of Autoimmunity with Systemic and Cutaneous Lupus Erythematosus**

**Authors:** Jingru Tian^1,2,3,4*†^, Liqing Shi^1,2,3†^, Dingyao Zhang^5,6^, Xu Yao^1,4^, Jun Lu^6,7,8,9,10^, Ming Zhao^1,2,3*^, and Qianjin Lu^1,2,3*^

* Correspondence should be addressed to:

Qianjin Lu, 12 Jiangwangmiao Street, Xuanwu, Nanjing, Jiangsu, China 210042.

Telephone: 025-85478999; Fax: 025-85414477;

Email: [qianlu5860@pumcderm.cams.cn](mailto:qianlu5860@pumcderm.cams.cn)

Ming Zhao, 12 Jiangwangmiao Street, Xuanwu, Nanjing, Jiangsu, China 210042.

Telephone: 025-85478999; Fax: 025-85414477;

Email: zhaoming307@126.com

Jingru Tian, 12 Jiangwangmiao Street, Xuanwu, Nanjing, Jiangsu, China 210042.

Telephone: 025-85478999; Fax: 025-85414477;

Email: [jingru.tian@pumcderm.cams.cn](mailto:jingru.tian@pumcderm.cams.cn)

**The PDF file includes:**

Figs. S1-6

Table. S1

Fig. S1.


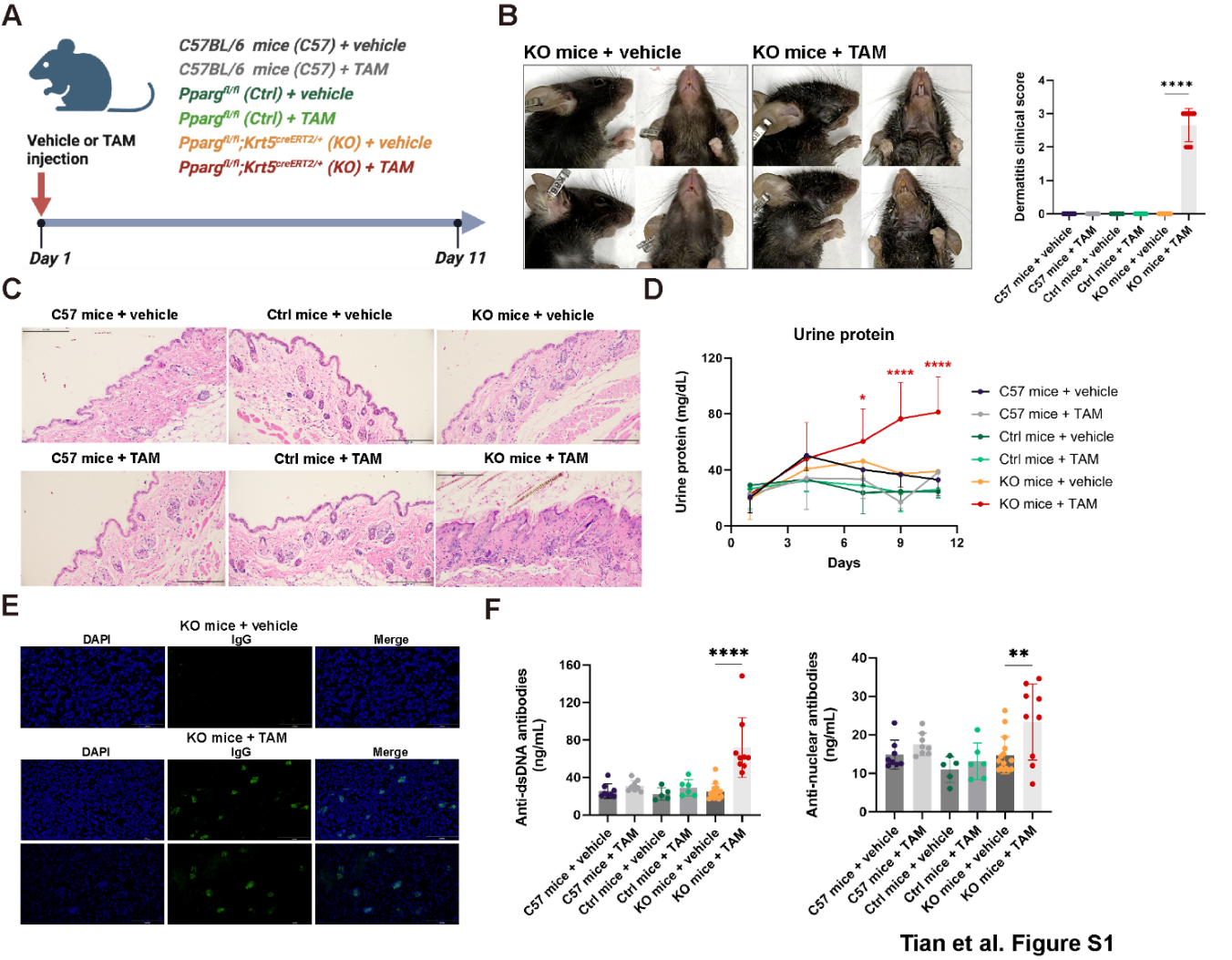


**Fig. S1. Keratinocyte-intrinsic PPARγ reduction drives the spontaneous development of an SLE-like phenotype in vivo.**

**(A)** Schematic illustration of the SLE model in which the level of keratinocyte-intrinsic PPARγ was decreased systemically. Tamoxifen (TAM) (1 mg per dose) or vehicle was administered intraperitoneally from Day 1 to Day 5 to C57BL/6 (C57), *Pparg*^fl/fl^ (Ctrl), and *Pparg*^fl/fl^;*Krt5*^creERT2/+^ (KO) mice. The kidney, skin and spleen were harvested on Day 11. **(B)** Representative images of mice from a representative experiment and graph showing dermatitis clinical scores. Images were acquired on the same day. **(C)** Representative images of the histological examination of prothorax skin on Day 11. **(D)** Urine protein levels were quantified on the indicated days after TAM or vehicle injection. (n = 5-15 per group). **(E)** Representative images of glomerular IgG deposition in mice on Day 11. **(F)** The levels of anti-double-stranded DNA antibody (left panel) and antinuclear antibody (right panel) in the peripheral blood of mice on Day 11. Error bars represent SDs, and center values indicate means. Two-tailed unpaired Student’s t-tests were used. **p* < 0.05; ***p* < 0.01; ****p* < 0.001; *****p* < 0.0001. Data are representative of two or more independent experiments.

Fig. S2.


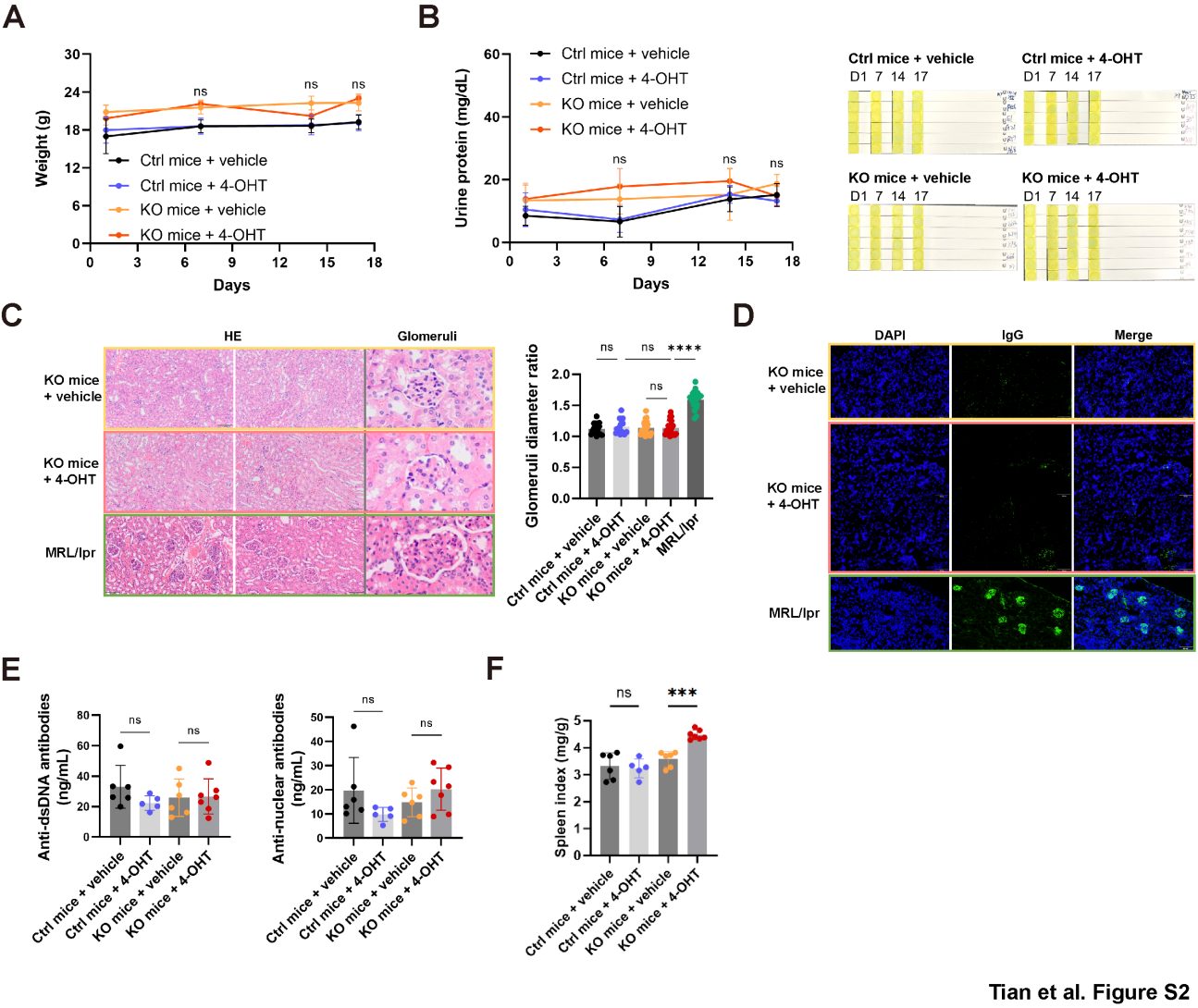


**Fig. S2. CLE-like phenotype in mice does not involve systemic manifestations**

**(A)** Body weight measured on indicated days (n = 6-11 per group). **(B)** Urine protein levels quantified (left) and qualified (right) on indicated days (n = 5-6 per group). **(C)** Representative histological images of kidneys and glomeruli, with a graph showing glomerular diameter ratio on Day 17. **(D)** Representative images of glomerular IgG deposition on Day 17. **(E)** Levels of anti-double-stranded DNA (left) and antinuclear antibodies (right) in peripheral blood on Day 17. **(F)**  Graph showing spleen index (spleen weight [mg] to body weight [g] ratio) (n=5-7). Error bars represent SDs, and center values indicate means. Comparisons between two groups were performed using a two-tailed unpaired Student's t-test, and multiple group comparisons were analyzed using one-way ANOVA. **p* < 0.05; ***p* < 0.01; ****p* < 0.001; *****p* < 0.0001; ns: not significant. Data are representative of two or more independent experiments.

Fig. S3.


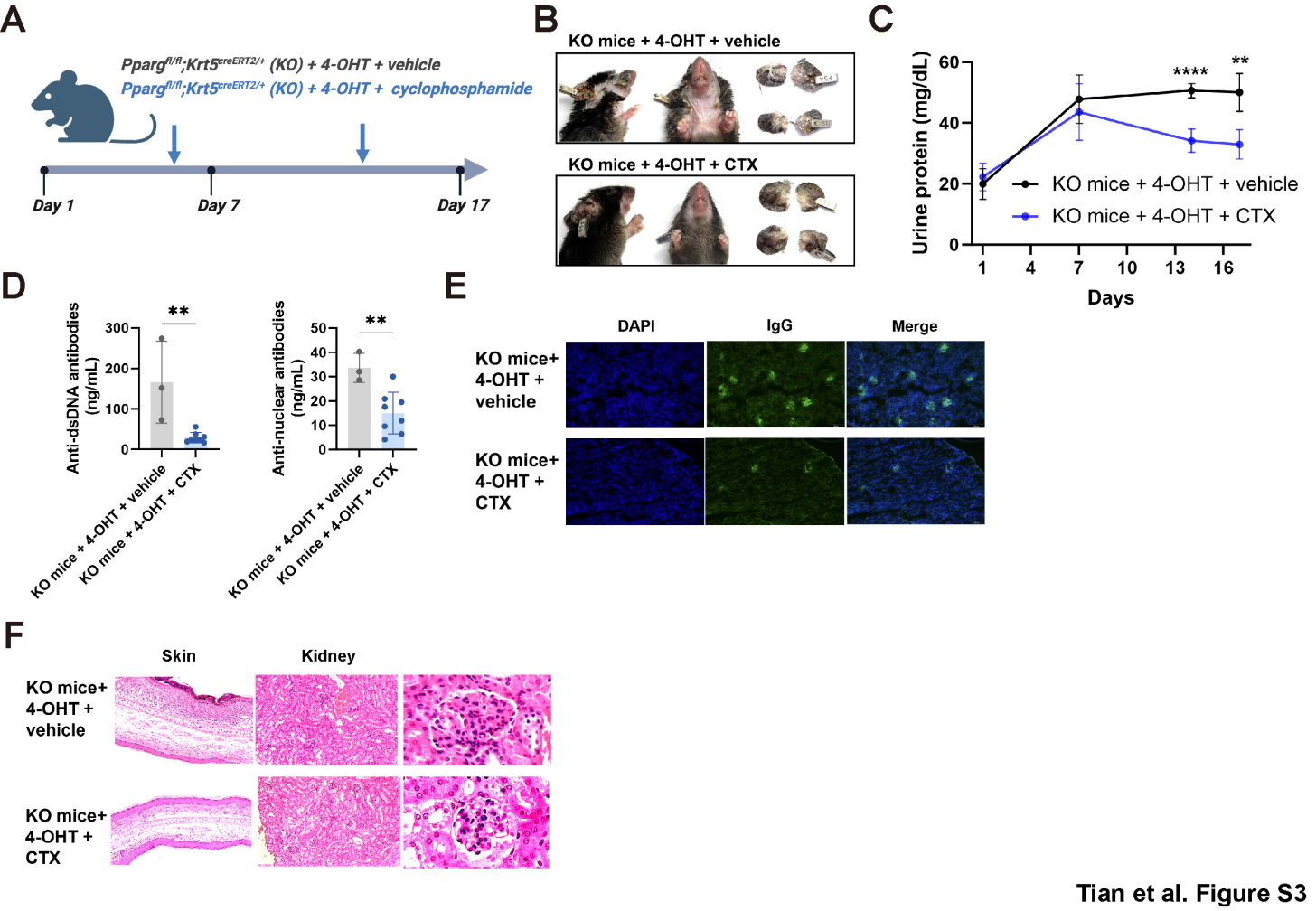


**Fig. S3. Cyclophosphamide treatment effectively alleviates the SLE-like phenotype**

**(A)** Schematic illustration of the SLE model treated with CTX via intraperitoneal injection. CTX (50mg/kg) or vehicle was administered intraperitoneally on Days 6 and 13, while 4-OHT was applied topically to both ears of *Pparg*^fl/fl^;*Krt5*^creERT2/+^ (KO) mice from Days 1 to 5. **(B)** Representative images of treated mice on Day 17. **(C)** Quantification of urine protein levels on indicated days (n = 3 or 8 per group). **(D)** Levels of anti-double-stranded DNA antibodies (left) and antinuclear antibodies (right) in peripheral blood (n = 3 or 8 per group). **(E)** Representative images of glomerular IgG deposition. **(F)** Representative histological images of skin, kidneys, and glomeruli. Error bars represent SDs, and center values indicate means. Two-tailed unpaired Student’s t-tests were used. **p* < 0.05; ***p* < 0.01; ****p* < 0.001; *****p* < 0.0001. Data are representative of two or more independent experiments.

Fig. S4.


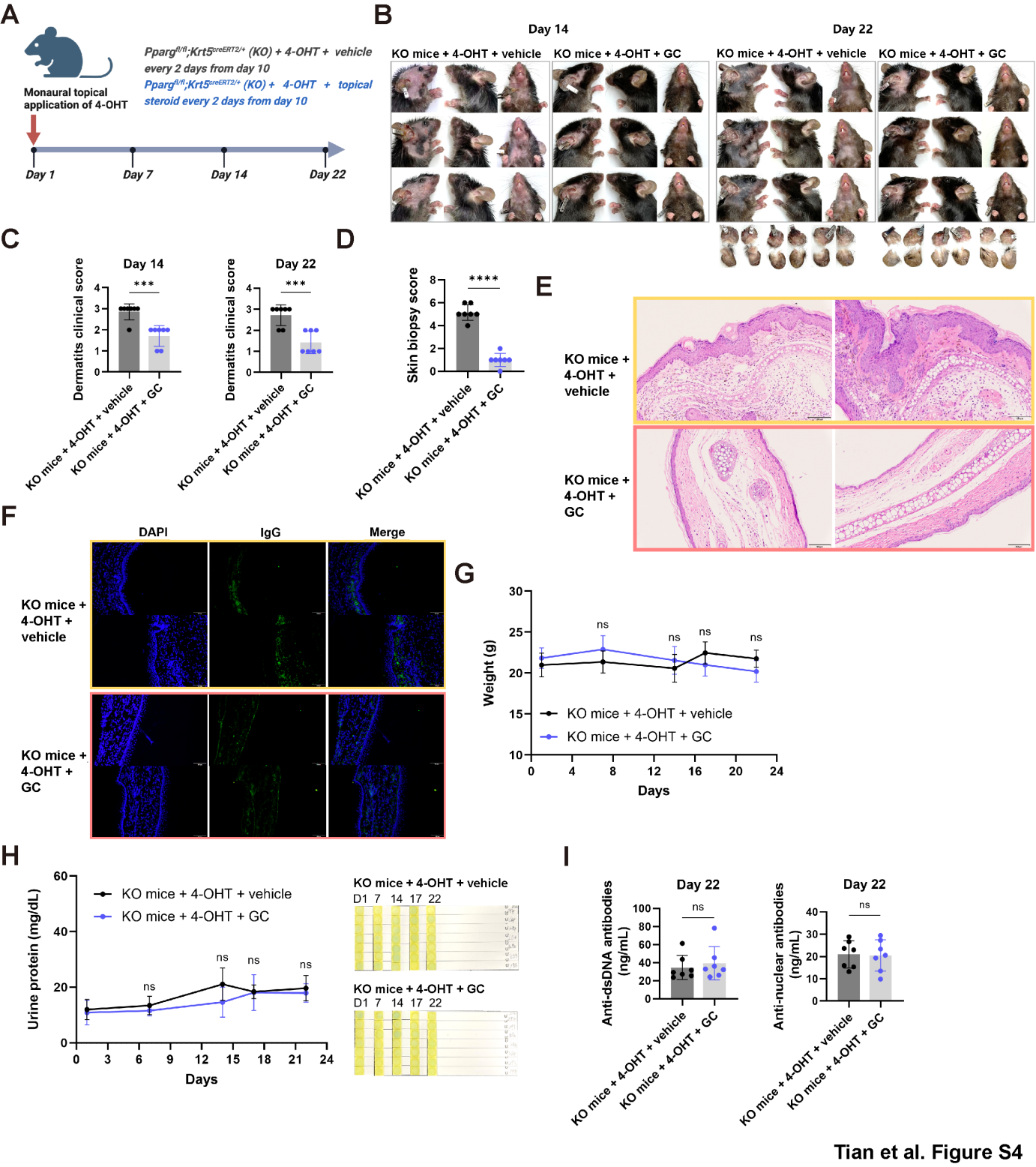


**Fig. S4. Topical steroid treatment effectively alleviates the CLE-like phenotype**

**(A)** Schematic illustration of the CLE model treated with GC topically. GC was applied topically to single ear of *Pparg*^fl/fl^;*Krt5*^creERT2/+^ (KO) mice every 2 days from Day 10, while 4-OHT was applied topically from Days 1 to 5. **(B)** Representative images of treated mice on Day 14 and Day 22. **(C)** Graph of dermatitis clinical scores on Days 14 and 22. **(D)** Graph of skin biopsy scores on Day 22. **(E)** Histological analysis of ear skin. **(F)** Images of IgG deposition in the skin basement membrane zone. **(G)** Body weight measured on indicated days (n = 6-11 per group). **(H)** Urine protein levels quantified (left) and qualified (right) on indicated days (n = 7 per group). **(I)** Levels of anti-double-stranded DNA antibodies (left) and antinuclear antibodies (right) in peripheral blood on Day 22 (n = 7 per group). Error bars represent SDs, and center values indicate means. Two-tailed unpaired Student’s t-tests were used. **p* < 0.05; ***p* < 0.01; ****p* < 0.001; *****p* < 0.0001; ns: not significant. Data are representative of two or more independent experiments.

Fig. S5.


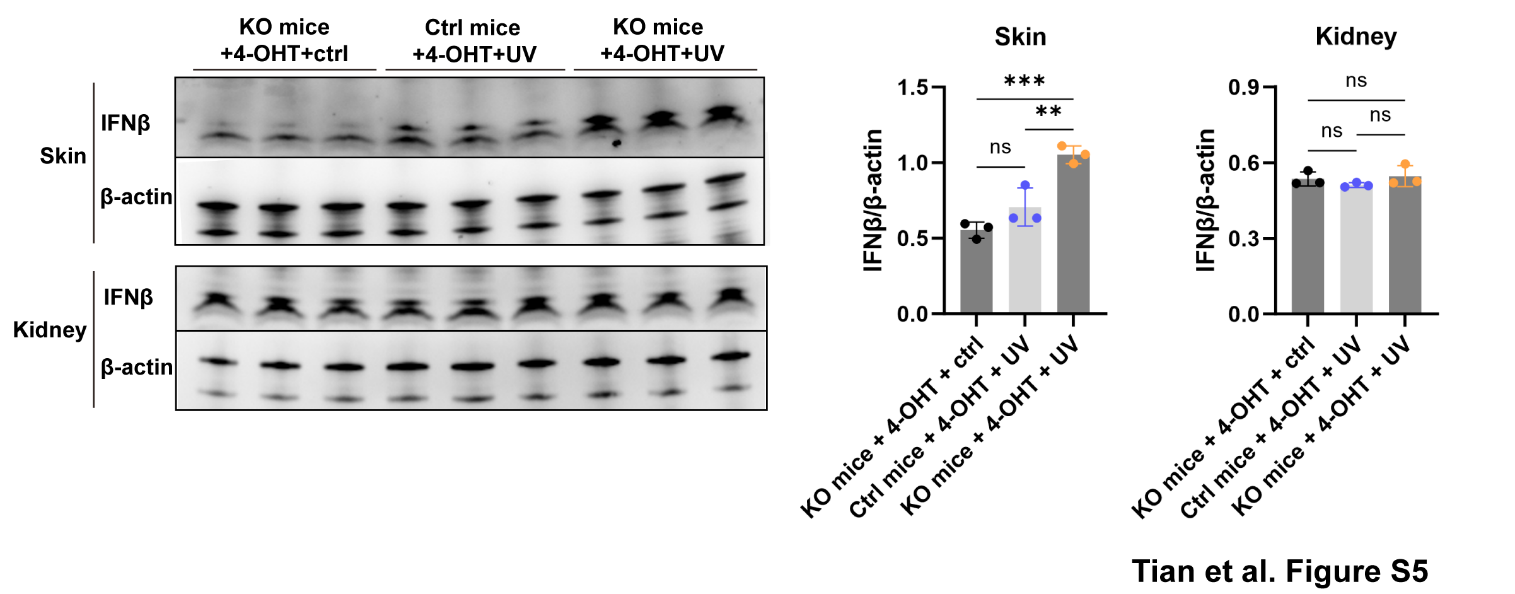


**Fig. S5. IFNβ protein level in skin and kidney tissues of mice.**

Representative western blot images showing IFNβ protein expression in skin lesions and kidney tissues and quantitative analysis of IFNβ protein levels normalized to β-actin (n = 3). Error bars represent SDs, and center values indicate means. Multiple group comparisons were analyzed using one-way ANOVA. **p* < 0.05; ***p* < 0.01; ****p* < 0.001; ns: not significant. Data are representative of two or more independent experiments.

Fig. S6.


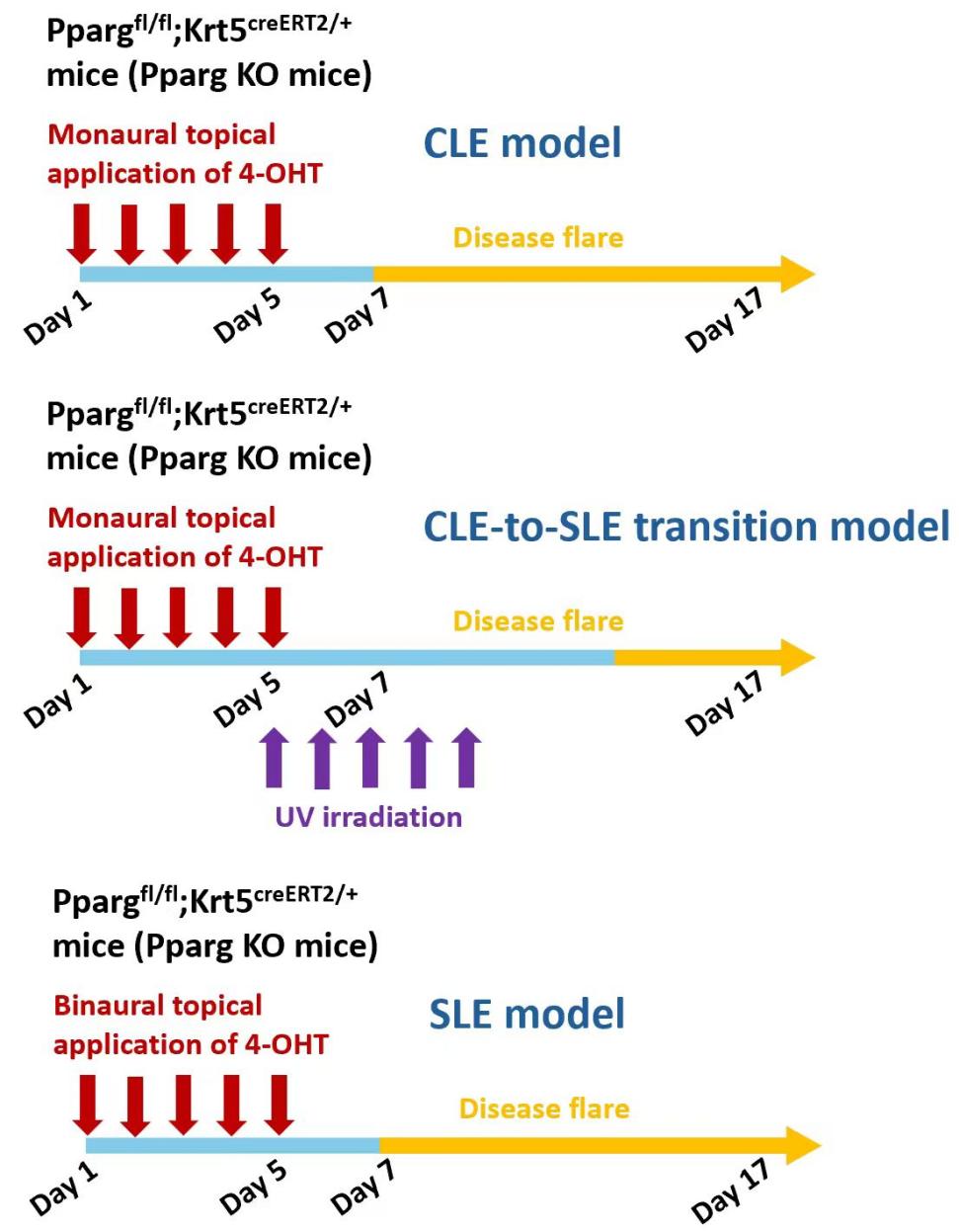


**Figure S6. Schematic flowchart of the experimental design for CLE, SLE, and induced SLE models.**

CLE model (top): *Pparg*^fl/fl^;*Krt5*^creERT2/+^ mice were treated with topical 4-OHT on one ear once daily for 5 consecutive days. Disease onset occurred approximately on day 7 after the initiation of 4-OHT application, and the mice remained in an active disease phase from day 7 to day 17. CLE-to-SLE transition model (middle): *Pparg*^fl/fl^;*Krt5*^creERT2/+^ mice were treated with topical 4-OHT on one ear once daily for 5 consecutive days, followed by daily UV irradiation for five days beginning on day 5 after 4-OHT application. From days 14 to 17, the mice developed systemic autoimmune phenotypes resembling SLE. SLE model (bottom): *Pparg*^fl/fl^;*Krt5*^creERT2/+^ mice were treated with topical 4-OHT on both ears once daily for 5 consecutive days. Disease onset occurred approximately on day 7 after the initiation of 4-OHT application, and the mice remained in an active disease phase from day 7 to day 17.

**Table S1. Clinical characteristics of patients and healthy volunteers.**

| No. | Gender | SLEDAI | CLASI | EASI | PASI | Age | Skin lesion location | Course of disease (years) |
| --- | --- | --- | --- | --- | --- | --- | --- | --- |
| SLE1 | Male | 6 | N/A | N/A | N/A | 58 | Face | 10 |
| SLE2 | Female | 7 | N/A | N/A | N/A | 49 | Upper limbs | 25 |
| SLE3 | Female | 5 | N/A | N/A | N/A | 36 | Face | 3 |
| SLE4 | Female | 10 | N/A | N/A | N/A | 41 | Scalp | 20 |
| SLE5 | Female | 6 | N/A | N/A | N/A | 54 | Upper limbs | 22 |
| SLE6 | Female | 5 | N/A | N/A | N/A | 32 | Upper limbs | 15 |
| CLE1 | Female | N/A | 8 | N/A | N/A | 34 | Face | 5 |
| CLE2 | Female | N/A | 6 | N/A | N/A | 41 | Face | 15 |
| CLE3 | Female | N/A | 6 | N/A | N/A | 29 | Upper limbs | 9 |
| CLE4 | Female | N/A | 5 | N/A | N/A | 33 | Face | 4 |
| CLE5 | Female | N/A | 11 | N/A | N/A | 39 | Scalp | 8 |
| AD1 | Male | N/A | N/A | 10 | N/A | 12 | Back | 2 |
| AD2 | Male | N/A | N/A | 8 | N/A | 20 | Upper limbs | 6 |
| AD3 | Female | N/A | N/A | 6 | N/A | 35 | Back | 5 |
| AD4 | Female | N/A | N/A | 25 | N/A | 14 | Back | 1 |
| AD5 | Female | N/A | N/A | 17 | N/A | 25 | Abdomen | 3 |
| AD6 | Female | N/A | N/A | 7 | N/A | 41 | Lower limbs | 4 |
| PSO1 | Male | N/A | N/A | N/A | 22 | 45 | Upper limbs | 25 |
| PSO2 | Male | N/A | N/A | N/A | 16 | 34 | Upper limbs | 18 |
| PSO3 | Male | N/A | N/A | N/A | 10 | 28 | Lower limbs | 9 |
| PSO4 | Female | N/A | N/A | N/A | 32 | 24 | Abdomen | 2 |
| PSO5 | Female | N/A | N/A | N/A | 9 | 16 | Abdomen | 4 |
| HC1 | Female | N/A | N/A | N/A | N/A | 34 | Face | N/A |
| HC2 | Male | N/A | N/A | N/A | N/A | 33 | Face | N/A |
| HC3 | Male | N/A | N/A | N/A | N/A | 24 | Face | N/A |
| HC4 | Female | N/A | N/A | N/A | N/A | 35 | Upper limbs | N/A |
| HC5 | Female | N/A | N/A | N/A | N/A | 31 | Scalp | N/A |
| HC6 | Female | N/A | N/A | N/A | N/A | 43 | Face | N/A |

SLEDAI: Systemic Lupus Erythematosus Disease Activity Index; CLASI: Cutaneous Lupus Erythematosus Disease Area and Severity Index; EASI: Eczema Area and Severity Index; PASI: Psoriasis Area and Severity Index; N/A: not applicable.
